# A pathogenic variant in the transforming growth factor beta I (*TGFBI*) in four Iranian extended families segregating granular corneal dystrophy type II: A literature review 

**DOI:** 10.22038/ijbms.2020.36763.8757

**Published:** 2020-08

**Authors:** Aliasgar Mohammadi, Azam Ahmadi Shadmehri, Mahnaz Taghavi, Gholamhossein Yaghoobi, Mohammad Reza Pourreza, Mohammad Amin Tabatabaiefar

**Affiliations:** 1 Department of Genetics and Molecular Biology, School of Medicine, Isfahan University of Medical Sciences, Isfahan, Iran; 2 Department of Genetics, Islamic Azad University, Science and Research Branch, Tehran, Iran; 3 Zeiss Ophthalmology Clinic, Tabas, South Khorasan, Iran; 4 Department of Ophthalmology, Birjand University of Medical Science, South Khorasan, Iran; 5 Social Detrimental Health Center, Birjand University of Medical Science, South Khorasan, Iran; 6 Pediatric Inherited Diseases Research Center, Research Institute for Primordial Prevention of Non-communicable Disease, Isfahan University of Medical Sciences, Isfahan, Iran

**Keywords:** Corneal dystrophy, Iran, Next-generation sequencing, Pathogenic variant, TGFBI

## Abstract

**Objective(s):**

Granular and lattice corneal dystrophies (GCDs & LCDs) are autosomal dominant inherited disorders of the cornea. Due to genetic heterogeneity and large genes, unraveling the mutation is challenging.

**Materials and Methods:**

Patients underwent comprehensive clinical examination, and targeted next-generation sequencing (NGS) was used for mutation detection. Co-segregation and *in silico* analysis was accomplished.

**Results:**

Patients suffered from GCD. NGS disclosed a known pathogenic variant, c.371G>A (p.R124H), in exon 4 of *TGFBI*. The variant co-segregated with the phenotype in the family. Homozygous patients manifested with more severe phenotypes. Variable expressivity was observed among heterozygous patients.

**Conclusion:**

The results, in accordance with previous studies, indicate that the c.371G>A in TGFBI is associated with GCD. Some phenotypic variations are related to factors such as modifier genes, reduced penetrance and environmental effects.

## Introduction

Granular and lattice corneal dystrophies (GCDs & LCDs) are heterogeneous autosomal dominant disorders. Gradual accumulation of hyaline, amyloid and non-amyloid deposits within anterior stromal layer of the cornea lead to decrement of sight acuity and visual impairment in the first or second decades of life (1). Mutations in at least nine genes *ARSC1, CHST6, COL8A2, GLA, GSN*, *KRT3,* *KRT12, M1S1* and *UBIAD1* have been reported in various types of CDs (2, 3). LCDs and GCDs are transforming growth factor beta induced protein (*TGFBI*)-linked corneal dystrophies and heterozygous mutations in *TGFBI* (OMIM 601692, previously called *BIGH3*), on human chromosome 5q31 is responsible for the disease (4). *TGFBI* mutations were first identified in human lung adenocarcinoma cell line by Skonier *et al* (5). For the first time, four missense mutations in the *TGFBI *gene were reported by Munier *et al.*, as causative gene in patients with four different types of CD (6). Until now, more than 70 various mutations including missense, non-sense, deletions and insertions have been reported to cause diverse types of CDs (7). Two hot spot codons in *TGFBI* in relation with LCDs and GCDs are R124 and R555 situated within exons 4 and 12, respectively (8). LCDs and GCDs are epithelial–stromal CDs according to the latest classification of International Committee for the Classification of Corneal Dystrophies (9). GCD type II (Avellino type or ACD; OMIM 607541), was first described by Felborg *et al.* in patients from Avellino origin, Italy (10). ACD is characterized as granular or combined granular-lattice, grayish-white, crumb-like, superficial and non-amyloid deposits accumulation within anterior third of corneal stroma and/or amyloid lattice opacities in deeper sites of cornea (11). The *TGFBI* gene encodes a 68-kDa extracellular matrix (ECM) containing 683 amino acid protein called Keratoepithelin (12). The TGFBI protein is expressed in different cell types as well as corneal stromal epithelium cells (13). The protein is involved in many cell processes and functions such as cell adhesion, cell migration, cell differentiation and autophagy phenomenon (14, 15). It has four Fasciclin like (FAS1) domains in C-terminus. Arginine 124 is situated within the first FAS1 domain, a conserved extracellular domain involved in cell adhesion interactions. Mutations lead to abnormal protein processing and accumulation (16). There are few reports of pathogenic variants in the TGFBI gene in Iranian population. In this study, we applied next-generation sequencing for molecular diagnosis of ACD in an extended Iranian kindred. A known pathogenic variant was co-segregating with the phenotype in the pedigree.

## Materials and Metods

***Subjects***

Four large isolated pedigrees from a village in South Khorasan province of Iran with several affected members suffering from visual problems were recruited. Precise clinical examinations including slit-lamp examination for available normal and affected members were performed by an ophthalmologist. A complete family history was obtained and the pedigrees were drawn by a medical geneticist. Subsequently, full investigation revealed familial relationship between these four selected pedigrees. After taking informed written consent, peripheral blood samples were obtained in EDTA-containing tubes.

***Molecular analysis***

Genomic DNA was extracted from peripheral blood lymphocytes using Prime Prep Genomic DNA Extraction kit (GeNet Bio, Korea) according to the manufacturer’s instruction. Qualitative and quantitative assessment of genomic DNA was checked using 1.2% agarose gel and Nanospec cube biophotometer (Nanolytik^®^, Dusseldorf, Germany).

 A custom designed Nimblegen chip was used to capture exons and exon-intron boundaries of the *TGFBI, UBIAD1, CHST6, VSX1, PIKFYVE, DCN, KRT12, and KRT3* genes and sequenced on an Illumina Hiseq 2000 in BGI-Clinical laboratories, Shenzhen, China. BWA was used for mapping short reads to the reference genome (hg19, NCBI Build 37), Picard for removal of duplicate reads and GATK for variant calling. Annotation was performed by ANNOVAR. Heterozygous missense, start codon change, splice site, stop gain, stop loss and nidel variants with MAF < 1%, were filtered in dbSNP version 137, 1000 genomes database, NHLBI GO exome sequencing project (ESP) and exome aggregation consortium (ExAC). We applied online software tools including MutationTaster2, FATHMM, SIFT and PolyPhen-2 to investigate *in silico* pathogenicity prediction of the missense variant. Candidate variant was investigated in the Human Gene Mutation Database (HGMD) and in the literature to seek the variant novelty and its association with a phenotype.

 Using forward: 5’ TCCCTCCTTCTGTCTTCTGC 3’ and reverse: 5’ CTCGGGGAAGTAAGGCAGTT 3’ primers, exon 4 of the *TGFBI* gene was amplified. PCR products were sequenced bi-directionally on an automated Genetic Analyzer ABI 3130XL (Applied Biosystems, Foster City, California, USA) using Big Dye Terminator Cycle Sequencing Kit (Applied Biosystems, Foster City, California, USA) and analyzed with Sequencer 5.4.5 (Gene Codes Corporation). Sequences were compared with reference sequence NM_000358. Variant nomenclature was based on HGVD and variant interpretation was according to the ACMG guideline. 

## Results

***Clinical and molecular findings***

We examined several affected and normal individuals of four pedigrees, aging 5 to 70 years. History of visual problems was found in more than 60 individuals that apparently were not close relatives. As a result of isolation condition, multiple consanguineous marriages were observed and affected children manifested with a more sever phenotype. A broad spectrum of disease symptoms were observed among affected individuals, from subclinical forms to severe and pure granular and mixed lattice-granular forms of the disease. Some individuals were found with recurrence of dystrophy after bilateral corneal transplantation. NGS revealed a known missense disease-causing variant, c.371G>A (p.R124H), within exon 4 of the *TGFBI* geneand it was confirmed by Sanger sequencing. *In silico* prediction tools revealed the disruptive effect of the variant (Table 1). 

***Pedigree A***

**Figure 1:**
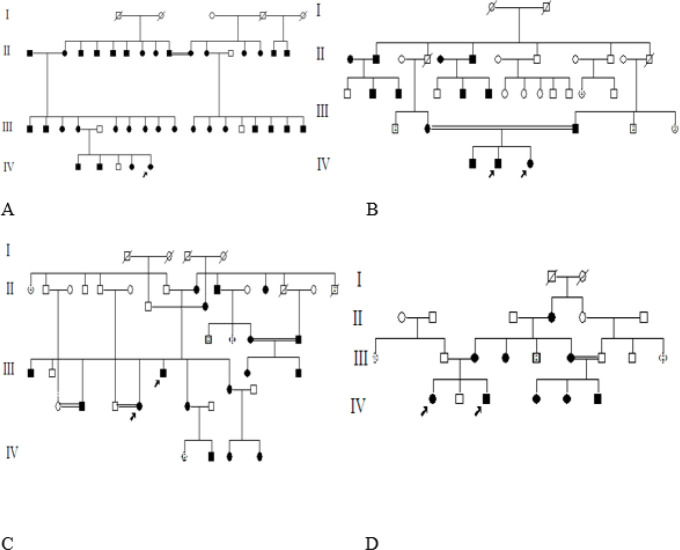
Pedigrees. Four large families were investigated. Several patients are available in each generation. Consanguineous marriages with affected individuals are common in the pedigrees, which make pattern of inheritance complicated in some cases

Proband was a 27-year-old female with light corneal dystrophy. She belonged to a large family with three generation history of visual impairment. The p.R124H variant was detected in the proposita, her mother and her affected siblings (Figure 1A). 

***Pedigree B***

**Figure 2:**
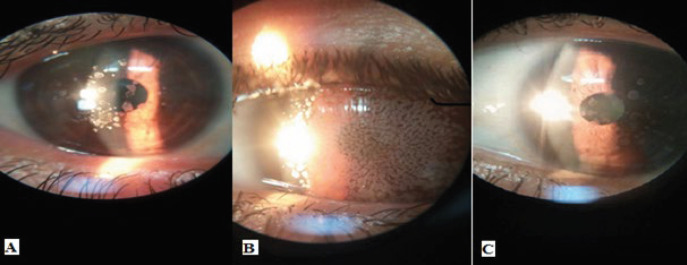
Slit-lamp photography. A: 5 years old girl shows granular deposits, B: the cornea of her 11 years old brother shows considerably dense granules. C: their 42 years old mother with no significant problem

Probands were two offsprings of a first cousin consanguineous marriage. A 5-year-old girl within a pedigree was diagnosed with mild, slowly progressive CD (Figure 2A). This condition decreased power of vision in both eyes to half at age 5. Slit-lamp examination showed granular deposits within cornea. Her 11-year-old brother was affected by more intensive form with considerable vision loss. He was diagnosed at age 6. The deposits were more aggregated than his 5-year-old sister (Figure 2B). The first offspring of the family was affected by milder form of ACD who was reported to be heterozygous for the mutation. Their 42-years-old mother was affected by a very milder form, with no visual impairment. Light scattering at night was the only problem for the mother. The size and density of deposits were considerably smaller than her offsprings (Figure 2C). Molecular results showed that parents were heterozygous. 

***Pedigree C***

Two mild form affected Probands referred to know about their offspring risk evaluation and genetic counseling. Several affected members were found in their maternal family with a wide spectrum of the disease phenotype. The heterozygous variant co-segregated with the phenotype in the pedigree (Figure 1C).

***Pedigree D***

A large association with individuals affected with CD in three generations was identified with mild form of the disease (1D). Sequencing results revealed heterozygous pathogenic variant, p.R124H, in all of these patients. Healthy individuals were carrying wild type alleles in homozygous status. 

**Table 1:** Table 1. In silico analysis of the variant pathogenicity for c.371 G>A in *TGFBI*

Software	MutationTaster2.0	SIFT	PolyPhen-2	FATHMM
Prediction	Disease causing	Damaging	Probably damaging	Damaging
Score	NA	0.022	0.958	-2.69

NA; Not Available

**Figure 3:**
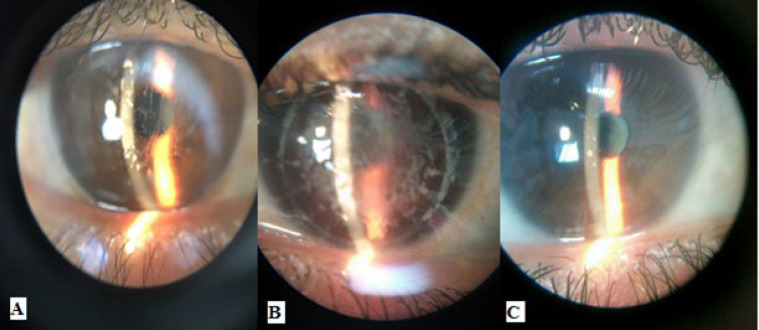
Slit-lamp photography, A: a patient with combined Lattice- Granular dystrophy, B: a patient with relatively dense granules, C: a patient with no significant problems

**Figure 4:**
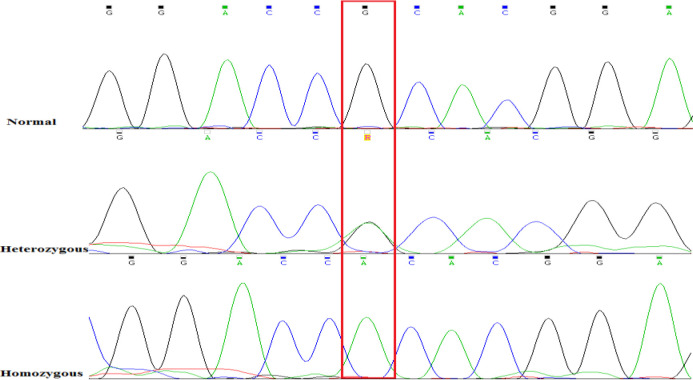
DNA sequences for pathogenic variant c.371 G>A (p.R124H) in exon 4. Above: homozygous normal variant, only G at position c.371, middle: Heterozygous state for the variant c.371 G>A, G and A peaks are visible (black and green respectively), below: homozygous pathogenic variant c.371 G>A

**Table 2:** Reported pathogenic variants in the TGFBI gene

	Coding Position	Protein alteration	Exon	Phenotype	Countries	Ref
1	c.337G>A	p.V113I	4	GCD	Mexico	[30]
2	c.367G>C	p.D123H	4	Atypical GCD, low penetrance,	Vietnam	[31]
3	c.370C>T	p.R124C	4	LCD1,TBCD,RBCD,GCD2	China, Korea, Japan	[6]
4	c.371G>A	p.R124H	4	GCD, GCD2	Japan, Korea, China, UK, Iran, Germany ,India, Hong Kong, …	[6]
5	c.371G>A; c.1631A>G	p.R124H;N544S	4;12	LCD1	Japan	[7]
6	c.371G>A; c.del307- 308delCT	p.R124H; NM	4	GCD	Hong Kong	[7]
7	c.371G>T	p.R124L	4	CDRB; Atypical GCD	India, Brazil, USA, Czech, China, France	[32]
8	c. 371 G > T ;ΔACGGAG	p.R124L ; ΔT125-ΔE126	4	FVGCD( Atypical GCD)	France	[33]
9	c.370C>T	p.R124S	4	GCD1	UK	[8]
10	c.393G>T	p.Glu131D	4	Schnyder Crystalline like CD phenotype (no mutation in UBIAD gene	Germany	[34]
11		p.A179*, p.R124H	4	GCD2	Korea	[35]
12	c.895G>A	p.D299N	7	Polymorphic LCD	USA	[7]
13	c.1486C>T	p.R496W	11	LCD IV	Japan	[7]
14	c.1501 C > A	p.P501T	11	LCDIIIA	China, Japan	[36]
15	c.1504 A > G	p.M502V	11	Unknown	Mexico	[37]
16	c.1504 A > G; c.1664 G>A	p.M502V;p.R555Q	11,12	Atypical TBCD	France	[7]
17	c.1514T>A	p.V505D	11	LCD I	China	[38]
18	c.1526 T>C	p.L509P	11	GCD2, LCD1	Germany, France	[7]
19	c.1526T>G	p.L509R	11	LCD1,EBMD	France	[7]
20	c.1541G>C	p.R514P	11	LCD	China	[39]
21	c. 1545T>A	p.F515L	11	LCD1	China	[39]
22	c.1548C>G	p.S516R	11	GCDI Like	India	[40]
23	c.1553T>C	p.L518P	12	LCD1 (early LCD)	Japan	[41]
24	c.1553T > G	p.L518R	12	LCD1/IIIA	Italy	[8]
25	c.1565 T > A	p.I522N	12	LCD I	China	[42]
26	c.1580T>G	p.L527R	12	LCDIIIA	Japan, Korea	[43]
27	c.1603G 4 T	G535T	12			[44]
28	c.1613 C > G	p.T538R	12	LCD1/IIIA	Ukraine, USA	[8]
29	c.1612A>C	p.T538P	12	LCD1	China, India	[7]
30	c.1616T>A	p.V539D	12	LCD	India	[16]
31	c.1618-1620delTTG	p.∆F540	12	CDLI/IIIA first reported as RBCD	Sardinia,	[8, 45]
32	c.1619T>C	p.F540S	12	LCDIII/A	Germany	[46]
33	c.1625C>G	p.P542R	12	LCD	Korea	[7]
34	c.1631A>G	p.N544S	12	LCD	Japan	[47]
35	c.1637 C > A	p.A546D	12	LCD,GCD, Polymorphic LCD	China, Mexico, India, USA, Germany	[48]
36	c.1637 C > A; c.1652 C > A	p.A546D;P551Q	12,12	LCD1	USA	[7]
37	c.1636G>A	p.A546T	12	LDCIIIA	Brazil ,China, France	[49]
38	c.1640 T> C	p.F547 S	12	Atypical LCD	Hungary	[50]
39	c.1645G>A;c.1663 C>T	p.A549T;R555W	12	GCD1	Germany	[7]
40	c.1649 C > T	p.L550P	12	GCDII	Mexico, Singapore	[37]
41	c.1649C>T;c.1877A>G	p.L550P;p. H626R	12,	Atypical GCD	Mexico	[7]
42	c.1652 C > A	p.P551Q	12	LCD	USA	[48]
43	c.1664 G>A	p.R555Q	12	CDTB, RBCD	Brazil, China, Czech, France, Japan, Singapore, Switzerland, Ukraine, USA	[6]
44	c.1663 C>T	p.R555W	12	GCD1, GCD2,RBCD	Brazil, China, Czech, Mexico, France, Japan, Singapore, Switzerland, Ukraine, USA, Taiwan, Turkey, Vietnam, New Zealand, India, Hong Kong, Spain, UK, Germany, Hungary	[6, 7]
45	c.1673T> C	p.L558P	12	LCDIII	Ukraine	[51]
46	c.1673T>G	p.L558R	12	LCD	Czech Republic	[52]
47	c.1675T>G	p.L559V	12	Atypical GCD	India	[40]
48	c.1694T> C	p.l565p	12	LCD	Poland	[53]
49	c.1706T>A	p.L569Q	13	LCD1	Korea	[35]
50	c.1753 T > G	p.L569R	13	LCD similar to distinct forms of type I	USA, Korea	[54]
51	c.1762 A > C	p.H572R	13	LCD1,LCDIIIA	Thailand, Singapore, Chile, China, Korea	[55]
52	c.1761_1763del	p.His572del	13	unilateral, late-onset variant of LCD	USA	[56]
53	c.1681G>T	p.G594V	13	late onset , deep stromal LCD	India	[16]
54	c.1838T>G	p.V613G	14	LCDIII	France	[7]
55	c.1837-1848del12bp GTTGCCGAGCCT	p.del613-616VAEP	14	LCD variant	China	[57]
56	c.1903 T> A	p.M619K	14	CGLCD	USA	[58]
57	c.1859G>A	p.A620D	14	Classic LCD	Singapore	[59]
58	c.1858C>G	p.A620P	14	LCDIIIA	Korea	[60]
59	c.1861C>A	p.T621P	14	LCDIIIA	Korea	[35, 61]
60	c.1866 T>G	p.N622K	14	CDLI/IIIA	Italy, South America	[8]
61	c.1864A>C	p.N622H	14	CDLI/IIIA	USA	[7]
62	c.1868 G > A	p.G623D	14	LCD, RBCD	Switzerland, China, USA, Germany	[8]
63	c.1867C>G	p.G623R	14	LCDI, LCDIIA	Germany	[62]
64	c.1870 G > A	p.V624M	14	Unilateral LCD		[63]
65	c.1874T>A	p.V625 D	14	Early onset of LCD	China	[64]
66	c.1870-1874del GTGGTC	p.del624-625VV	14	Atypical LCD	India	[16]
67	c.1877A>G; c.1618-1620delTTG	p.H626R; ΔF540	14	CDLI/IIIA	N M	[8]
68	c.1924 A > C	p.H626P	14	CDLI/IIIA, RBCD, TBCD	New Zealand, Czech	[8]
69	c.1877 A>G	p.H626R	14	Asymmetric LCD I/III	China, Mexico, France, Singapore, Ukraine, Vietnam, India, UK, Germany,	[59]
70	[1885 1886ins9(CCAATGTTC)]	p.(NVP629–630ins)	14	LCD III/A	France	[65]
71	c.1939 T > A	p.V631D	14	LCD III/A	Italy	[8]
72	c.1998G>C	p.R666S	16	EBMD	Ireland	[66]
73	c.1926 del G	premature truncation at amino acid 669	14	CDLI/IIIA	NM	[8]
74	c.598A>T	p.I200F	5	NM	NM	[7]
75	c.805C>T	p.L269F	7	NM	NM	[7]
76	c.1486C>G	p.R496G	11	NM	NM	[7]
77	c.337G>A c.1673T>C	p.V113I;p.L558P	4;12	LCD Variant	USA	[67]
78	c.370C>T; c.1408C>T	R124C;G470X*	4	LCD	Japan	[68]

GCD: Granular Corneal Dystrophy; LCD: Lattice Corneal Dystrophy; TBCD: Thiel-Behnke Corneal Dystrophy; RBCD: Reis Bukler Corneal Dystrophy; FVGCD: France Variant Corneal Dystrophy; CGLCD: combined granular-lattice corneal dystrophy; NM: Not Mentioned

* Presented data are arranged based on RefSeq NM_000358; NP_000349

## Discussion

Here we report a disease-causing variant, c.371G>A (p.R124H), at exon 4 of the *TGFBI *gene, in four large Iranian pedigrees. This position is considered as a hotspot codon in *TGFBI* (17). The variant affects the first FAS1 domain of the protein, probably by altering protein solubility and stability (18). Although mutations in TGFBI are distributed throughout the gene, there are four exons with the highest rate of missense mutations including exons 12, 14, 4 and 11, respectively (Table 2). Indeed, TGFBI-linked CDs are the great examples for genotype-phenotype correlation. Specific mutation leads to a specific outcome; furthermore, special mutations in TGFBI-linked CDs are found to be related to the disease severity regardless of homozygous or heterozygous status (18). It seems that mutations in primary exons, especially within exon 4, have more contribution to create granular types of corneal dystrophies and middle exons, especially exon 12 are more responsible for lattice types (Table 2). R124H has the most contribution among GCD2 cases. Mashima and colleagues reported GCD2 patients with p.R124H mutation (19). Alavi and colleagues reported a group of patients affected with GCD2 and reported p.R124H in Iranian population and Middle East for the first time. Here we report the largest Iranian group of GCD2 patients with more than 70 affected individuals from four pedigrees living in an isolated village. It seems that GCD2 is the most frequent type of the disease in Iranian population, and p.R124H is considered as the most common cause of the disease (20, 21). More investigations are needed to evaluate the prevalence of *TGFBI* mutations in CD Iranian patients. The phenotype of homozygous patients were more severe than heterozygous individuals, as earlier age of onset, rapid progression of the disease or more deposits within the cornea in concordance with previous studies (22). Because all patients were sharing the same mutation, we suppose that founder mutation or genetic drift are the responsible mechanisms for high disease prevalence in this isolated village. Clinical variability observed among heterozygous individuals is in concordance with previous investigations (23, 24). However, the reasons of this phenomenon are not completely understood; we hypothesized this results from the effect of modifier genes and other loci on expression of the *TGFBI* gene. Reduced penetrance, complexity of monogenic traits, epistasis interactions and environmental factors can be other explanations (25-27). Despite enormous advances in genetics, medicine and technology, there are rare successful treatments for monogenic disorders like CDs. In fact, performing procedures such as laser-assisted in situ keratomileusis (LASIK) for GCD2 patients can precipitate the course of the disease (28). We did not find history of LASIK in our patients, although it has a rare indication in such patients. Corneal transplantation had been operated for two of our patients, but disease manifestations were observed few years later in both of them (29).

## Conclusion

High frequency of *TGFBI* mutation, p.R124H, in Iranian population can result from a founder mutation or genetic drift. The results are useful for genetic counseling, cascade screening and prenatal diagnosis to reduce disease burden as there is not any treatment for the disease right now. 
